# Supplementary material for: Display of Microbial Glucose Dehydrogenase and Cholesterol Oxidase on the Yeast Cell Surface for the Detection of Blood Biochemical Parameters
Source: Biosensors (Basel). 2020 Dec 30;11(1):13. doi: 10.3390/bios11010013 (PMC7823397; doi:10.3390/bios11010013)
Supplement: Supplementary file 1 [file biosensors-11-00013-s001.pdf]

Supplementary Materials

Display of Microbial Glucose Dehydrogenase and Cholesterol Oxidase on the Yeast Cell Surface for the Detection of Blood Biochemical Parameters

Shiyao Zhao [1,†], Dong Guo [2,†], Quanchao Zhu [1], Weiwang Dou [1], and Wenjun Guan [1,*]

[1] Institute of Pharmaceutical Biotechnology and The Children's Hospital, Zhejiang University School of Medicine, Hangzhou 310012, China
[2] College of Pharmaceutical Sciences, Zhejiang University, Hangzhou 310012, China
* Correspondence: guanwj@zju.edu.cn; Tel.: +86-0571-88206477
† These authors contributed equally to this work.

Table S1. Strains and vectors used in this study.

Strains or Vectors	Description	References
**Vectors**		
pYD1	The vector containing a *GAL1* promoter and an *Aga2* gene, used in the *S. cerevisiae* a-agglutinin surface display system (RRID: Addgene_73447)	[1]
pYD1-GDH1	pYD1 derived with an insertion *GDH1* gene	This study
pYD1-CHO1	pYD1 derived with an insertion *CHO1* gene	This study
pYD1-CHO1-PASx1	pYD1-CHO1 derived with a modified linker which added one PAS sequence to the original GS linker	This study
pYD1-CHO1-PASx2	pYD1-CHO1 derived with a modified linker which added two PAS sequences to the original GS linker	This study
**Strains**		
E. coli TG1	Host strain for vector construction	[2]
S. cerevisiae EBY100	The strain with genomic insertion of an *AGA1* gene regulated by a *GAL1* promoter, used in the *S. cerevisiae* a-agglutinin surface display system (Genotype: *MAT*a *AGA1*::*GAL1-AGA1*::*URA3* *ura3-52* *trp1* *leu2*Δ*1* *his3*Δ*200* *pep4*::*HIS3* *prb1*Δ*1.6R* *can1* *GAL*)	[3]
P1	The *S. cerevisiae* EBY100 strain containing vector pYD1	This study
G1	The *S. cerevisiae* EBY100 strain containing vector pYD1-GDH1	This study
C1	The *S. cerevisiae* EBY100 strain containing vector pYD1-CHO1	This study
C2	The *S. cerevisiae* EBY100 strain containing vector pYD1-CHO1-PASx1	This study
C3	The *S. cerevisiae* EBY100 strain containing vector pYD1-CHO1-PASx2	This study

Table S2. Comparison of different cholesterol oxidases reported previously.

Source	Molecular weight (kDa)	Activity ($U \cdot mg^{-1}$)	Temperature stability	pH stability	Metal ions stability	Organic solvents stability	Detergents stability	Reference
Chromobacterium sp. DS-1 (Cho1 used in this study was cloned from this strain)	58	16.7	4-85℃	3.0-11.0	No metal ions are associated with the activity	Stable in various solvents (e.g., isopropanol, ethyl acetate, butanol, chloroform, benzene, toluene, *p*-xylene, cyclooctane) except acetone	Stable in Tween 20, Triton X-100, Triton X-405, sodium cholate at 30℃	[4]
Brevibacterium sterolicum ATCC 21387	59	17	\	\	\	\	\	[5]
Streptoverticillium cholesterolicum	56	21.1	\	4.0-12.5	Activity inhibition by Hg^{2+}, Ag^{+}, Fe^{3+}, and Cu^{2+}	Activity inhibition in *p*-nitrophenol, *N*-bromosuccinimide, and 1-fluoro-2,4-dinitrobenzene	\	[6]
Rhodococcus equi No. 23	56	4.5	35-55℃	6.0-9.0	\	\	\	[7]
Streptomyces sp. SA-COO	55	23	\	\	\	Inactive in acetone, isopropanol, ethyl acetate, and butanol	Stable in Tween 20, Triton X-100, sodium cholate at 30°C	[8]

Pseudomonas sp. ST-200	60	15.2	4-50℃	4.0-11.0	\	Stable in various solvents except acetone and chloroform	\	[9]
Burkholderia cepacia ST-200	59	16.9	4-50℃	4.0-11.0	\	Stable in various solvents except acetone	Stable in Tween 20, Triton X-100, sodium cholate at 30°C	[10]
Pseudomonas aeruginosa	60	11.6	4-70℃	5.5-11.0	Activity inhibition by Ag^{+} and Cu^{2+}	Stable in various solvents except acetone and isopropanol	Stable in Tween 20, Triton X-100, Triton X-405, sodium cholate at 30℃	[11]
Castellaniella sp. COX	59	15	25-50℃	6.0-8.5	Activity inhibition by Hg^{2+}, Ba^{2+}, Fe^{2+}, Cu^{2+}, and Zn^{2+}	Stable in various solvents except acetone and ethyl acetate	Stable in Tween 20, Tween 40, Tween 60, Tween 80, Triton X-100, sodium cholate at 40°C	[12]

Table S3. Accuracy evaluation of the glucose biosensor.

Target glucose concentration (mg/dL)	YSI measured glucose concentration (mg/dL)	Average Current (μA)	Calculated glucose concentration (mg/dL)	Accuracy deviation
25	24.47	1.65	29.29	+4.82
50	47.82	1.69	57.09	+9.27
100	113.99	1.73	86.42	-24.19%
200	165.70	1.95	191.81	+15.76%
300	268.57	2.13	251.31	-6.43%
400	318.06	2.30	318.74	+0.21%
500	463.19	2.51	447.06	-3.48%
600	554.94	2.66	604.81	+8.99%
700	650.02	2.74	715.19	+10.03%
800	801.82	2.73	700.20	-12.67%

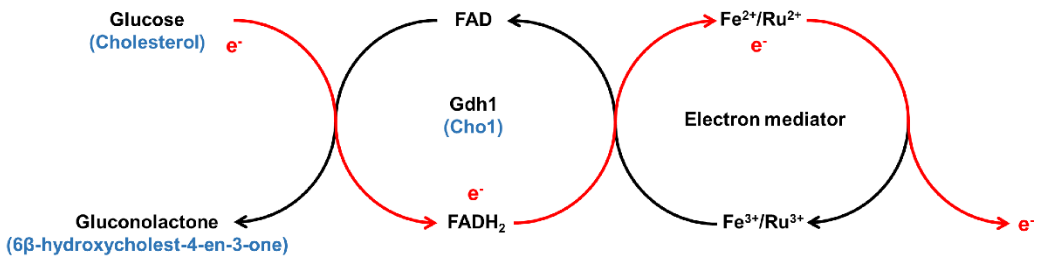

Figure S1. The detection mechanism of glucose or cholesterol biosensor. By the redox role of Gdh1 (or Cho1) immobilized on the electrodes, the intramolecular electron was transferred from glucose (or cholesterol) to the coenzyme (FAD) of Gdh1 (or Cho1). Then the electron was captured by the redox mediator mixed with the enzymes. With the redox mediator switched from reduced state to oxidized state under the action of an applied voltage, the electron was transferred from the redox mediator to the working electrode [13]. Ultimately, the working electrode of biosensor read the current which was related to the amount of glucose or cholesterol in the sample.

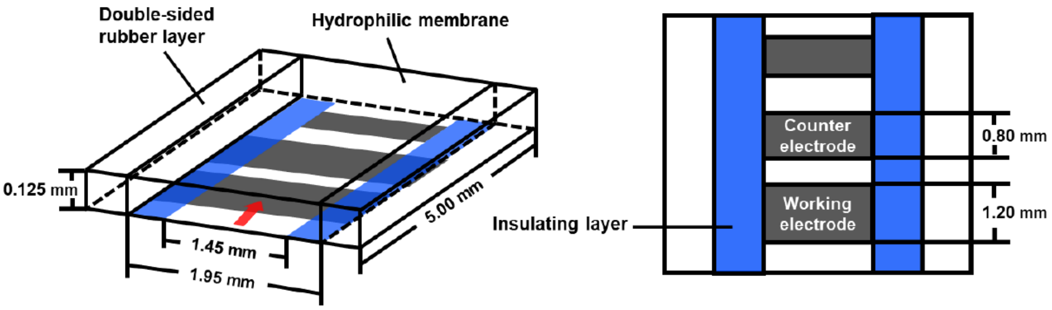

Figure S2. Schematic diagram of the reaction chamber of the electrode strips.

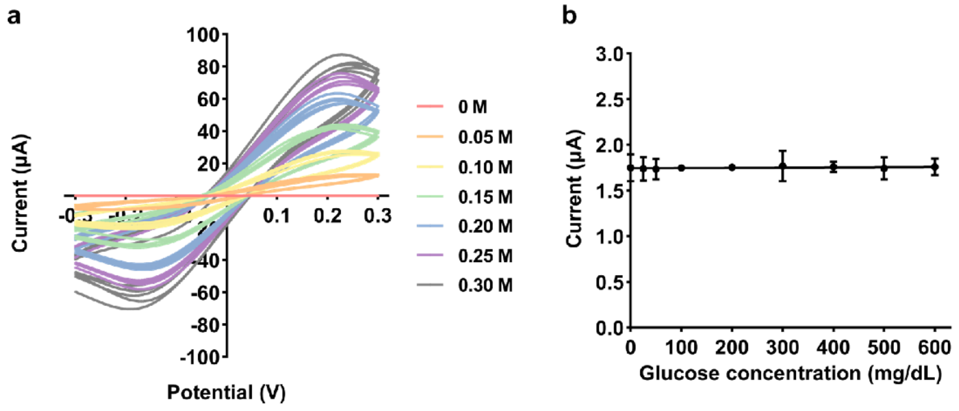

Figure S3. Performance evaluation of the screen-printed carbon electrodes. (a) Cyclic voltammetry curves of the blank screen-printed carbon electrodes with different concentrations of $K_3[Fe(CN)_6]/K_4[Fe(CN)_6]$. Five repetitions are shown for each concentration. (b) The response currents of the cell-free screen-printed carbon electrodes dotted with FAD (0.6%) and hexaammineruthenium (III) chloride (6.5%) to different glucose concentrations in whole blood samples. Error bars indicate the SD of samples tested in triplicate.

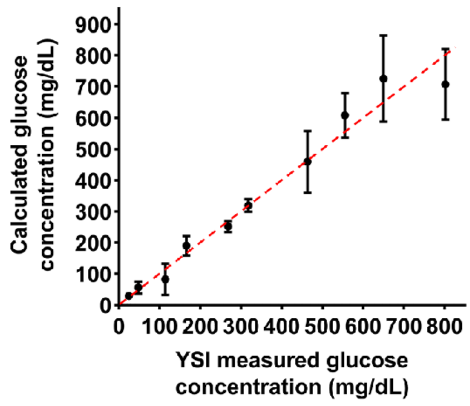

Figure S4. Accuracy evaluation of the glucose biosensor. According to the fitting relationship between glucose concentration measured by YSI and response current read by the biosensor, a third-order polynomial equation, $y=891.37x^3-5543.5x^2+11819x-8386.7$, was obtained. Then the current values were substituted into equation, corresponding glucose concentrations were calculated. The red dashed line represented a standard line to evaluate the degree of deviation of the biosensor detection result. Error bars indicate the SD of samples tested in triplicate.

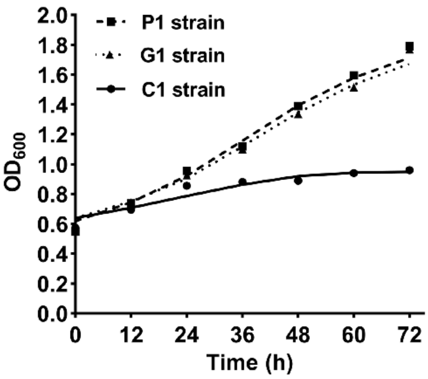

Figure S5. Growth curve of the P1, G1 and C1 strains in the induction medium containing 2% galactose. Each data point represents the mean value of three replicates.

References

1. Kieke, M.C.; Cho, B.K.; Boder, E.T.; Kranz, D.M.; Wittrup, K.D. Isolation of anti-T cell receptor scFv mutants by yeast surface display. *Protein engineering* **1997**, *10*, 1303-1310.
2. Gibson, T. Studies on the Epstein-Barr virus genome. University of Cambridge, 1984.
3. Boder, E.T.; Wittrup, K.D. Yeast surface display for screening combinatorial polypeptide libraries. *Nature biotechnology* **1997**, *15*, 553-557.
4. Doukyu, N.; Shibata, K.; Ogino, H.; Sagermann, M. Purification and characterization of *Chromobacterium* sp. DS-1 cholesterol oxidase with thermal, organic solvent, and detergent tolerance. *Applied microbiology and biotechnology* **2008**, *80*, 59.
5. Uwajima, T.; Yagi, H.; Nakamura, S.; Terada, O. Isolation and crystallization of extracellular 3β-hydroxysteroid oxidase of *Brevibacterium sterolicum* nov. sp. *Agricultural and biological chemistry* **1973**, *37*, 2345-2350.
6. Inouye, Y.; Taguchi, K.; Fujii, A.; Ishimaru, K.; Nakamura, S.; Nomi, R. Purification and characterization of extracellular 3*β*-hydroxysteroid oxidase produced by *Streptoverticillium cholesterolicum*. *Chemical and pharmaceutical bulletin* **1982**, *30*, 951-958.
7. Watanabe, K.; Aihara, H.; Nakagawa, Y.; Nakamura, R.; Sasaki, T. Properties of the purified extracellular cholesterol oxidase from *Rhodococcus equi* No. 23. *Journal of agricultural and food chemistry* **1989**, *37*, 1178-1182.
8. Ishizaki, T.; Hirayama, N.; Shinkawa, H.; Nimi, O.; Murooka, Y. Nucleotide sequence of the gene for cholesterol oxidase from a *Streptomyces* sp. *Journal of bacteriology* **1989**, *171*, 596-601.
9. Doukyu, N.; Aono, R. Purification of extracellular cholesterol oxidase with high activity in the presence of organic solvents from *Pseudomonas* sp. strain ST-200. *Applied and environmental microbiology* **1998**, *64*, 1929-1932.
10. Doukyu, N.; Aono, R. Cloning, sequence analysis and expression of a gene encoding an organic solvent-and detergent-tolerant cholesterol oxidase of *Burkholderia cepacia* strain ST-200. *Applied microbiology and biotechnology* **2001**, *57*, 146-152.
11. Doukyu, N.; Nihei, S. Cholesterol oxidase with high catalytic activity from *Pseudomonas aeruginosa*: Screening, molecular genetic analysis, expression and characterization. *Journal of bioscience and bioengineering* **2015**, *120*, 24-30.
12. Devi, S.; Sharma, B.; Kumar, R.; Singh Kanwar, S. Purification, characterization, and biological cytotoxic activity of the extracellular cholesterol oxidase produced by *Castellaniella* sp. COX. *Journal of basic microbiology* **2020**, *60*, 253-267.
13. Suzuki, N.; Lee, J.; Loew, N.; Takahashi-Inose, Y.; Okuda-Shimazaki, J.; Kojima, K.; Mori, K.; Tsugawa, W.; Sode, K. Engineered glucose oxidase capable of quasi-direct electron transfer after a quick-and-easy modification with a mediator. *International journal of molecular sciences* **2020**, *21*, 1137.
